# Early Experience Comparing Reduced Planning Target Volume Margins Using Cone Beam Computed Tomography Scan Guided Online Adaptive Radiation Therapy to Nonadaptive, Traditional Margin Plans for Muscle-Invasive Bladder Cancer

**DOI:** 10.1016/j.adro.2025.101934

**Published:** 2025-10-24

**Authors:** Samuel B. Hayworth, Whitney S. Hotsinpiller, Joel Pogue, Melissa Tyler, Joseph Harms, Carlos Cardenas, Dennis Stanley, Andrew McDonald

**Affiliations:** a Department of Radiation Oncology, The University of Alabama at Birmingham Heersink School of Medicine, Birmingham, Alabama; b Department of Radiation Oncology, Washington University School of Medicine, St. Louis, Missouri

## Abstract

**Purpose:**

Radiation therapy (RT) for muscle-invasive bladder cancer (MIBC) requires substantial planning target volume (PTV) margins to accommodate bladder-filling variability. We hypothesized that cone beam computed tomography (CBCT) scan guided online adaptive (AD) RT (oART) improves target coverage while reducing normal tissue exposure compared with non-AD RT.

**Methods and Materials:**

Over the course of a year, 8 patients with MIBC received oART. Five patients received 55 Gy in 20 fractions to a clinical target volume (CTV) that was expanded from the transurethral resection of a bladder tumor bed and 46 Gy to the remaining bladder; in contrast, 3 patients had the entire bladder designated as the high-dose CTV. PTVs were 7 mm isometric expansions of CTVs for AD treatment and non-AD (scheduled [SC]) treatment plans versus 15 mm for conventional large margin (LM) treatment plans. Target coverage and organ-at-risk (OAR) dosimetry for AD treatment plans were compared with SC and LM treatment plans per fraction using the Wilcoxon paired test. Total treatment time and acute toxicities were assessed.

**Results:**

The AD treatment plan was selected for the delivery of all fractions. Daily bladder volumes differed from simulation by a mean of 60 (SD, ± 66) cc. The CTV D98% > 98% was met for 160 (100%) of fractions with AD treatment plans versus 140 (87.5%) for LM and 67 (41.9%) with SC treatment plans. The CTV_high V90% = 100% for all AD treatment plans. Besides rectum_V30, AD treatment plans reduced OAR exposure for all metrics. Target and OAR objectives were met for 62.3%, 55.4%, and 91.2% of SC, LM, and AD treatment plans, respectively. Median fraction time was 24.7 minutes. Acute toxicity included only 3 grade 1 toxicity events and 2 grade 2 genitourinary or gastrointestinal toxicity events, with no occurrences of grade 3 or higher.

**Conclusions:**

oART achieved acceptable dosimetry for target volumes with a reduced PTV margin of 7 mm, despite considerable daily bladder volume variation. AD treatment plans improved target coverage and OAR dosimetry. Future patient-centered studies should explore the long-term clinical impact of reduced margin of oART for MIBC.

## Introduction

Primary chemoradiation therapy (CRT) is an alternative to radical cystectomy for many patients with muscle-invasive bladder cancer (MIBC) who wish to preserve their bladder or are not operative candidates. The clinical target volume (CTV) for bladder-preserving radiation therapy (RT) consists of the entire bladder with the option to include draining lymphatics and a partial bladder boost. To account for variability in bladder volume and other intrafraction and interfraction variability, planning target volume (PTV) margins of up to 1.5 cm are required to ensure adequate target coverage even with daily image guidance (IG) with cone beam computed tomography (CBCT) scan.^1^, ^2^, ^3^ Large PTV expansions result in greater normal tissue exposure, particularly concerning for radiosensitive small bowel. Modern treatment techniques, such as intensity modulated RT (IMRT), reduce radiation exposure to normal tissues outside of the PTV but cannot address overlap between the target and small bowel or interfractional anatomic variation.

Online adaptive (AD) RT (oART) is an emerging RT technique that allows for daily modification of IMRT plans to account for interfractional anatomic variation. Using daily IG and adapting contours and plans to real-time anatomy, PTV margins can be reduced significantly.^1^^,^^4^, ^5^, ^6^ Prior in silico studies suggest that oART may improve the therapeutic ratio for MIBC by improving target coverage and reducing organ-at-risk (OAR) exposure; however, clinical reports comparing reduced PTV margin oART to conventional large margin (LM) treatment plans remain lacking.^1^^,^^7^, ^8^ Although oART may widen the therapeutic index, it is resource- and time-intensive.^9^^,^^10^

The goal of this study was to report our institution’s early experience implementing oART in the treatment of MIBC. Primarily, we compared pertinent clinical dosimetric endpoints to our oART plans versus scheduled (SC) non-AD treatment plans. We further add to the clinical literature by also providing a comparison of AD treatment plans to standard image guided radiation therapy with conventional LM treatment plans. Lastly, we provided total treatment times, our institution’s oART workflow, and acute toxicity.

## Methods and Materials

This institutional review board-approved single institution retrospective cohort study includes consecutive patients with non-metastatic MIBC who completed definitive RT to the bladder and adjacent structures using oART alone since our oART program began in November 2021 to December 2022. One patient treated with elective nodal irradiation (ENI) was excluded. All patients underwent maximal transurethral resection of bladder tumor (TURBT) before beginning RT.

### Simulation and contouring

Computed tomography (CT) scan simulation was performed immediately after voiding, with 2 to 3 mm slices from the L1 vertebral body to 4 cm below the ischial tuberosity. A comprehensive description of PTVs is given in Table 1. The high-dose CTV consisted of the TURBT bed expanded by 1 cm along the bladder mucosa or the entire bladder if the TURBT bed was poorly visualized or an extensive tumor was suspected. The low-dose CTV always included the entire bladder and was extended to include the prostate or proximal ureters, depending on tumor location. PTV expansions were 7 mm for the bladder and TURBT bed, and 5 mm for the prostate.

**Table 1:** Planning target volume (PTV) definition for each patient with the associated group stage

	5500 cGy volume		4600 cGy volume		Stage
SIB Utilized	Structures	PTV nomenclature	Structures	PTV nomenclature	
No SIB	Bladder	PTV			II
	Bladder and prostate	PTV			IIIa
	Bladder and prostate	PTV			IIIa
SIB	Bladder	PTV_high	Prostate	PTV_low	II
	Bladder	PTV_high	Prostate	PTV_low	II
	Bladder	PTV_high	Prostate and distal ureters	PTV_low	IIIa
	TURBT bed and distal L ureter	PTV_high	The remaining bladder and the portion of the remaining L ureter	PTV_low	II
	TURBT bed and node	PTV_high	Remaining bladder	PTV_low	IIIa

*Abbreviations:* SIB = simultaneous integrated boost; TURBT = transurethral resection of a bladder tumor.

### Treatment planning

AD and SC treatment plans were generated and delivered using Varian Ethos (Varian Medical Systems) according to the workflow outlined by Stanley et al.^11^^,^^12^ Autocontours were edited as needed, allowing a synthetic CT (sCT) scan to be generated based on a deformable image registration of the simulation CT scan and daily CBCT scan. The sCT scan was used for optimization/calculation, allowing SC and AD treatment plans to be generated by recalculating the initial treatment plan and reoptimizing a new treatment plan, respectively. The treating physician was present for 1 treatment per week and performed offline review for the remaining treatments, whereas a rotating team of AD physicists performed contour editing and treatment plan selection for other fractions.^13^ Conventional image guided radiation therapy/LM treatment plans treating only the PTV_high (bladder) with PTV expansions of 1.5 cm were generated in Ethos. These treatment plans were exported to Eclipse, and 3 degrees of freedom rigid registrations of the initial CT scan anatomy to daily CBCT scan anatomy (using target and local bony anatomy) allowed LM treatment plans to be emulated. Treatment plans were optimized to meet target coverage and OAR constraints (Table 2)**.** All analysis was performed in Eclipse using the native scripting API. Statistical comparisons were performed using the Wilcoxon paired nonparametric test with Bonferroni correction, with significance set at *P* < .00625. Reference treatment plan quality assurance (LM, AD, and SC treatment plans) for all patients demonstrated minimum ArcCheck pass rates of 96% (3%/2 mm), secondary dose calculation agreement of 99.9% (5%/3 mm), and mean PTV dose differences < 1.3%. No AD treatment fractions were rejected because of quality assurance failure.

**Table 2:** Planning objectives and compliance for target coverage and organ-at-risk (OAR) constraints for large margin (LM), scheduled (SC), and adaptive (AD) treatment plans with associated planning priority

Planning objective compliance			Priority	Treatment plan type		
				LM	SC	AD
Target	PTV	PTV_high D98% > 5225 cGy	1	140/160 (87.5%)	40/160 (25%)	157/160 (98.1%)
		PTV_low D98% > 4370 cGy	1	-	52/100 (52%)	100/100 (100%)
	CTV	CTV_high D98% > 5390 cGy	1	140/160 (87.5%)	67/160 (41.9%)	160/160 (100%)
		CTV_low D98% > 4508 cGy	1	-	95/100 (95%)	100/100 (100%)
OARs	Bowel	Bowel _003 cc < 5600 cGy	1	27/160 (16.8%)	105/160 (65.6%)	143/160 (89.4%)
		Bowel V5500 cGy < 3 cc	1	59/160 (36.9%)	132/160 (82.5%)	149/160 (93.1%)
		Bowel_V4500 cGy < 50 cc	2	87/160 (54.4%)	123/160 (76.9%)	138/160 (86.3%)
	Rectum	Rectum_Dmean < 2250	2	53/160 (33.1%)	106/160 (66.3%)	148/160 (92.5%)
		Rectum_V30 < 45%	2	115/160 (71.9%)	145/160 (90.6%)	160/160 (100%)
All objectives		Target + OARs	-	1/160 (0.6%)	11/160 (6.9%)	117/160 (73.1%)

Fraction numbers and corresponding compliance proportions for each metric are denoted under the plan type. Plan optimization was stratified by 2 priority levels, with priority 1 constraints higher in the cost function than priority 2.

*Abbreviations:* CTV = clinical target volume; PTV = planning target volume.

Patients were evaluated weekly throughout oART and at 1 month following completion of treatment. Acute toxicity was graded using the National Cancer Institute Common Terminology Criteria for Adverse Events version 5.0 criteria.

## Results

### Patient characteristics

Eight patients with MIBC were treated with definitive CRT using oART IMRT. Anatomic staging was conducted at the time of maximal TURBT, with 50% of patients having stage II and the remaining 50% having stage III disease. In total, 87.5% of patients underwent maximum TURBT before consultation, and 1 patient declined tumor debulking surgery. Our population ranged from 70 to 86 years of age, with an average of 78 ± 5.8 years. The majority (87.5%) were male. Most patients (75%) were White, and 25% were Black. Clinical performance status was assessed for each patient, with Eastern Cooperative Oncology Group scores ranging from 0 to 2; specifically, 37.5% of patients had a score of 0, 25% had a score of 1, and 37.5% had a score of 2.

### Treatment summary

**Figure 1:**
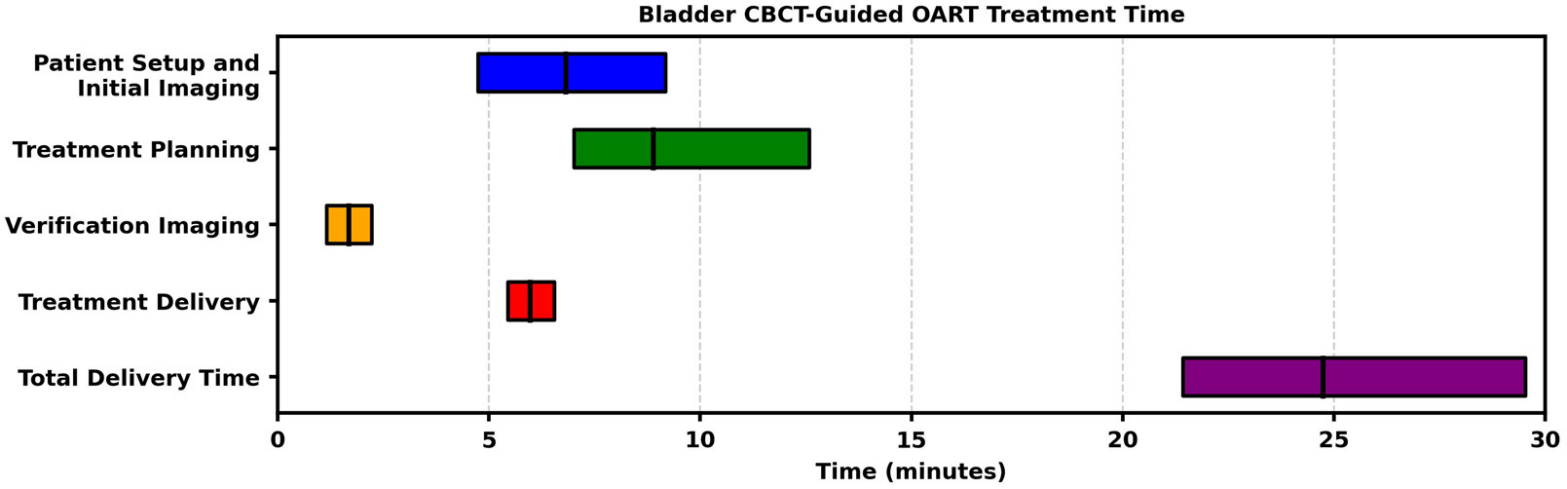
Box and whisker plots summarizing treatment times for bladder cone beam computed tomography (CBCT) scan guided online adaptive radiation therapy (oART).

All patients received hypofractionated RT in 20 fractions with concurrent systemic therapy. Systemic regimens were chosen by each patient’s medical oncologist and were based on patient-specific factors. Regimens mainly included 5-fluorouracil and mitomycin (62.5%), with the remaining patients (37.5%) receiving concurrent and/or neoadjuvant regimens consisting of pembrolizumab (12.5%), capecitabine and mitomycin (12.5%), or capecitabine alone (12.5%). Each patient completed their prescribed radiation treatment course in full, totaling a total of 160 fractions across our population. The AD treatment plan was selected for each treatment fraction by the same attending radiation oncologist, who was present once weekly and otherwise reviewed offline, whereas a rotating team of AD treatment physicists performed contour edits and plan selection for the remaining fractions; all 160 fractions’ plans were included for analysis. Median (IQR) time from TURBT to CT scan simulation was 29 (13-113) days, and time from simulation to first AD treatment plan was 26 (14-35) days. Daily bladder volumes differed from the bladder volume at simulation by a mean of 60 (SD, ± 66) cc. The average time to complete the daily AD treatment plan, from patient setup to treatment delivery, was 26.17 (SD, ± 6.75) minutes. The median time for treatment planning (autocontouring, contour editing, plan calculation and optimization, and plan selection) was 8.88 (4.92-25.85) minutes. The median time to complete verification imaging was 1.7 minutes. The median treatment delivery time was 5.97 minutes (Fig. 1).

### Dosimetric comparison

**Figure 2:**
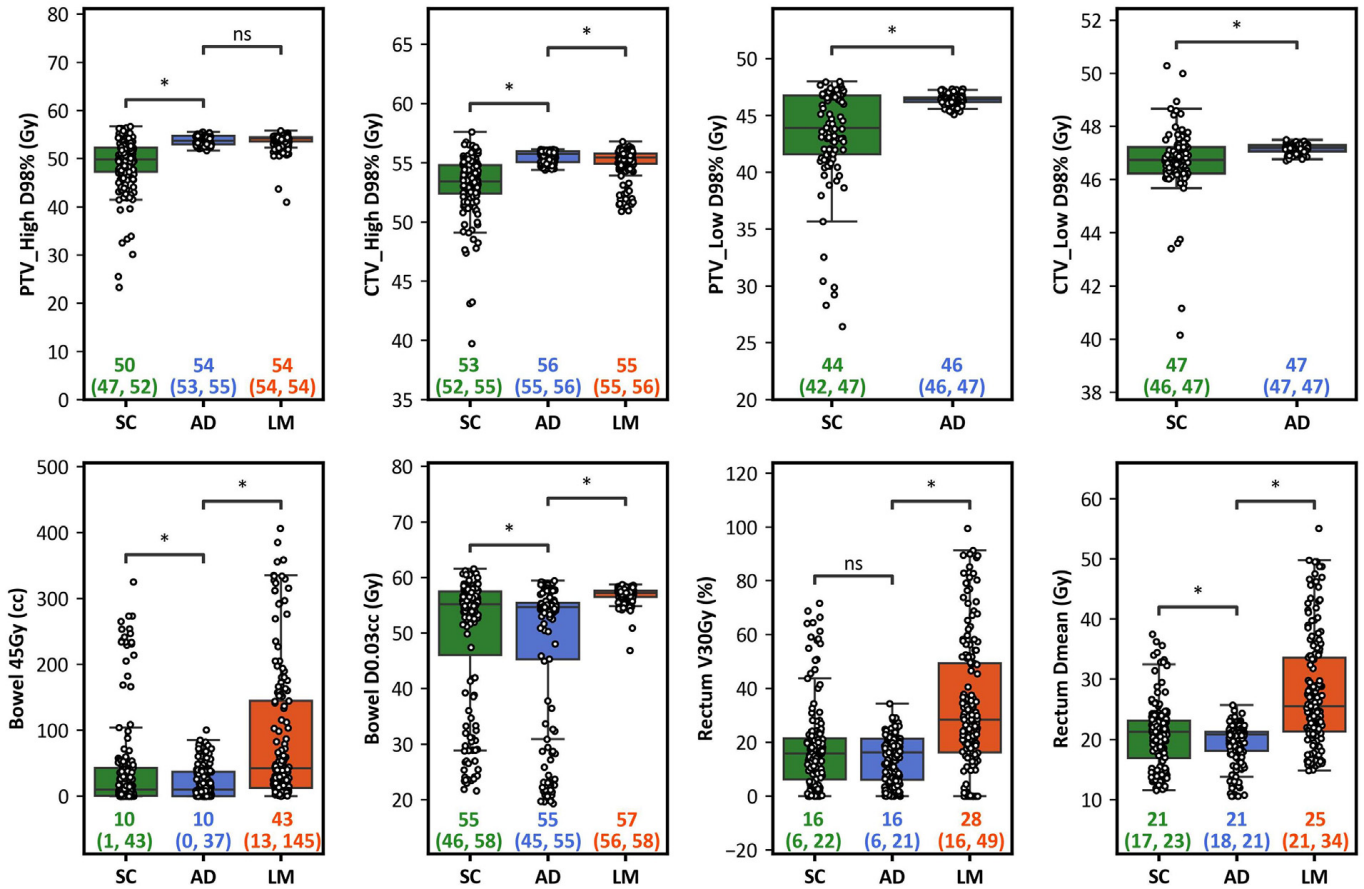
Box and whisker plots summarizing scheduled (SC, n = 160), adaptive (AD, n = 160), and large margin (LM, n = 160 fractions) treatment plans for each planning goal. The white circles demonstrate individual data points, boxes/lines represent interquartile ranges (IQRs)/medians, and whiskers illustrate data within 1.5 IQR of Q1/Q3. Median (Q1, Q3) target coverage and organ-at-risk (OAR) dosimetric parameters for AD, SC, and LM treatment plans are illustrated and compared using the Wilcoxon paired nonparametric test. *P < 0.00625. ns = nonsignificant.

Boxplots illustrating all LM, SC, and AD treatment plan data are shown in Fig. 2. Across both the PTV and CTV high and low volumes at the D98% dose constraint, the scheduled metric distributions were significantly inferior to the AD treatment plan metric distributions when comparing the median dose (*P*-value < .00625). This was consistent at the CTV_high volume when comparing the traditional IMRT LM treatment plans to the AD treatment plan, but no significant difference was observed at the PTV high volume (*P*-value = .498). For OARs, AD therapy plans delivered significantly lower bowel doses, as reflected by bowel_45 Gy and bowel_D0.03 cc, compared with SC and LM treatment plans. The AD treatment plan delivered significantly lower rectal doses compared with the LM treatment plan at both the rectum_V30 and rectum_Dmean metrics. The AD treatment plan also provided significantly lower doses than the SC treatment plan for rectum_Dmean, but no significant difference was observed between the AD and SC treatment plans at the rectum_V30 constraint. The average ± SD bowel_D003 cc for the SC, AD, and LM treatment plans were 4828.7 ± 1250.9, 4628.0 ± 1377, and 5667.3 ± 142.9 cc, respectively. The average ± SD bowel_V45 Gy for the SC, AD, and LM treatment plans were 42.9 ± 80.2, 17.3 ± 23.4, and 76.2 ± 106.1 cc, respectively. The average ± SD rectum_Dmean for the SC, AD, and LM treatment plans were 20.8 ± 5.5, 19.2 ± 3.42, and 27.6 ± 9.3 Gy, respectively. The average ± SD rectum_V30 Gy for the SC, AD, and LM treatment plans were 18.0 ± 16.1, 14.2 ± 8.6, and 33.0 ± 24.8 cc.

**Figure 3:**
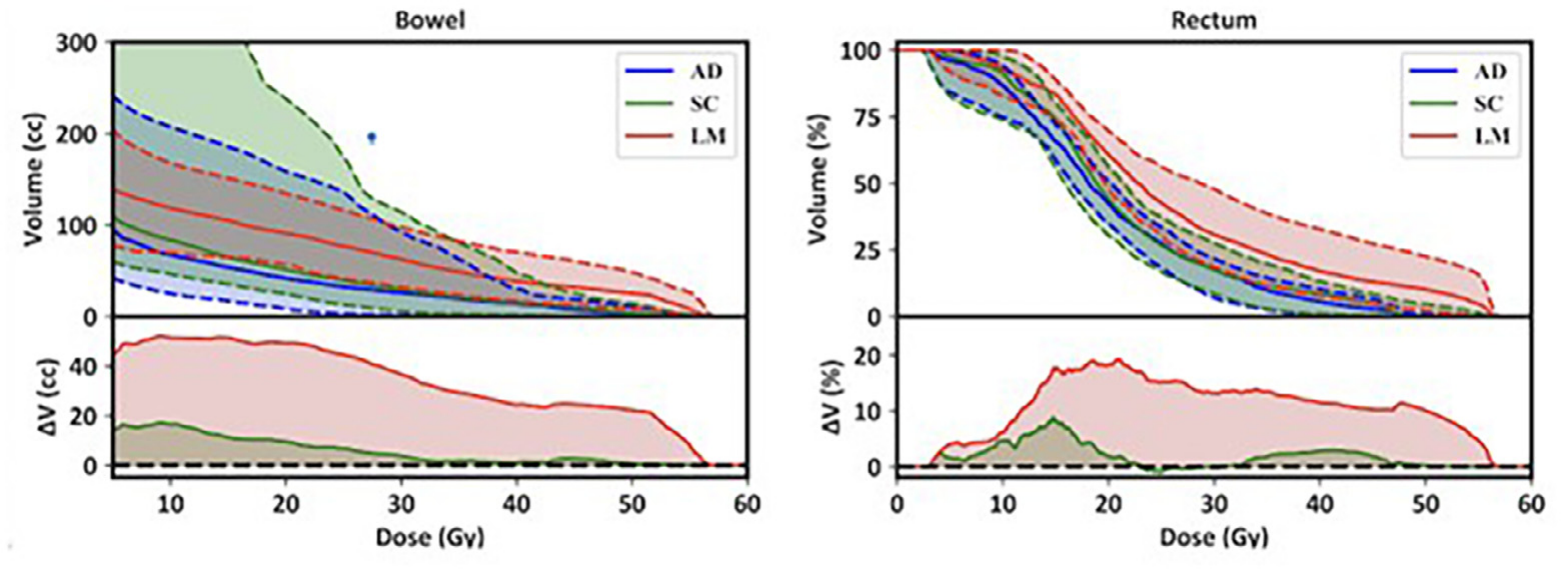
Median (Q1-Q3) adaptive (AD), scheduled (SC), and large margin (LM) treatment plans dose-volume histograms (DVHs) for bowel and rectum among all 160 fractions (top). Median DVH differences between AD treatment plans and SC/LM treatment plans are illustrated by the green/red shaded areas (bottom).

These differences are further demonstrated using dose-volume histograms (DVHs) in Fig. 3, where a reduction in AD treatment plan rectum and bladder volumes receiving most dose levels is observed, relative to SC and LM treatment plans.

**Figure 4:**
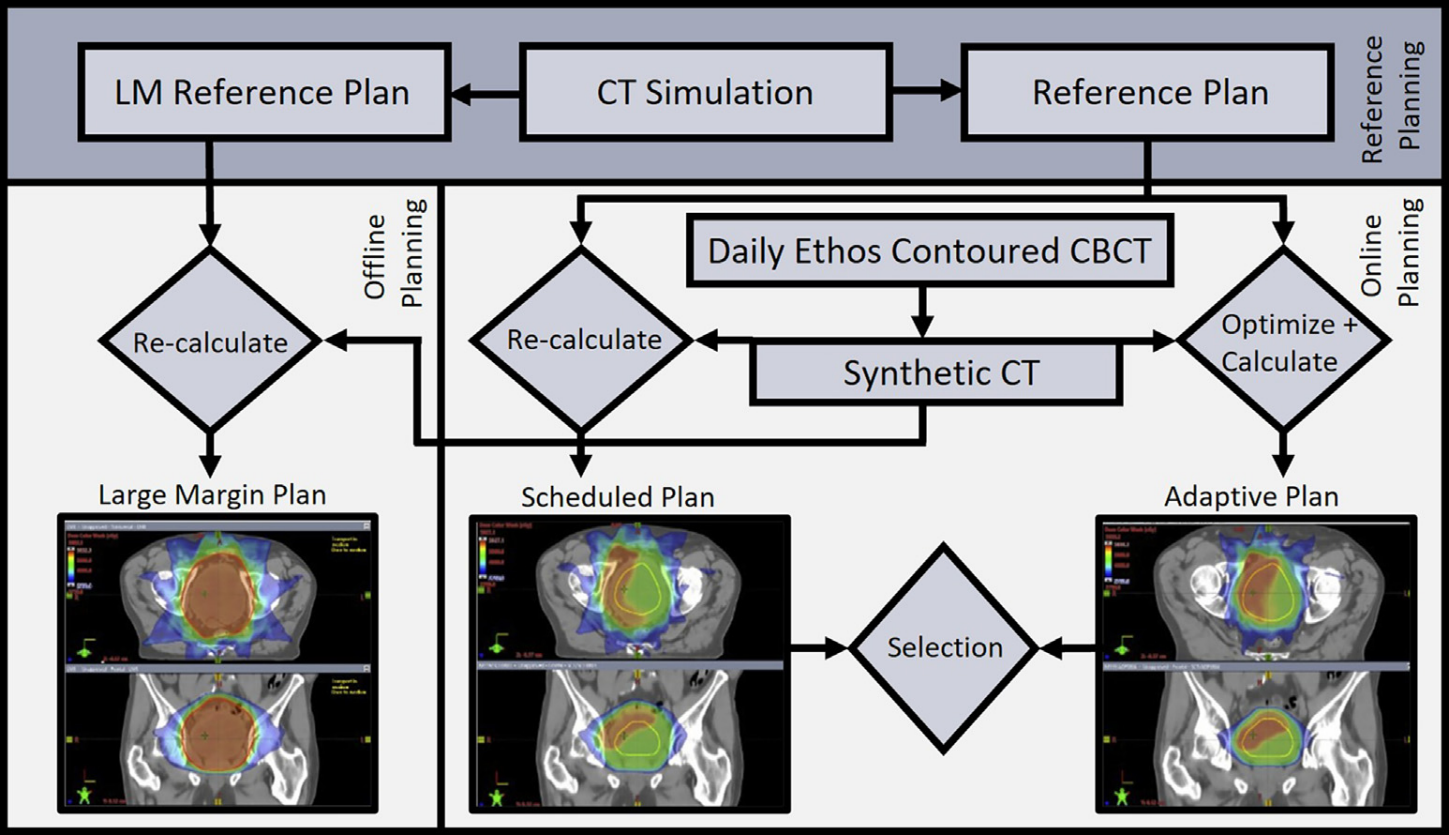
Workflow using Ethos and Eclipse for the generation of scheduled (SC), adaptive (AD), and large margin (LM) treatment plans. Autocontours from the daily cone beam computed tomography (CBCT) scan were edited as needed, allowing a daily synthetic CT (sCT) scan to be generated using a deformable image registration of the simulation CT scan to the daily CBCT scan. Ethos then generated SC and AD treatment plans for each fraction by recalculating the respective reference plan onto daily anatomy and optimizing a new plan using daily anatomy, respectively. Reference 1.5 cm LM treatment plans treating only the planning target volume (PTV)_high were generated on the CT scan simulation anatomy. These plans were then registered to a daily sCT scan using bony anatomy and recalculated in Eclipse for daily LM treatment plan generation.

Planning objective compliance for target coverage and OAR constraints is shown in Table 2. At least 98% of the high-dose CTV was covered by 98% of the prescription dose for 160 (100%) fractions with the AD treatment plan, compared with 140 (87.5%) fractions with the LM treatment plan and 67 (41.9%) fractions with the SC treatment plan. Target and OAR planning objectives were met for 62.3%, 55.4%, and 91.2% of SC, LM, and AD treatment plans, respectively. AD treatment plans met all compliance objectives in 73.1% of fractions (117/160), compared with 6.9% (11/160) for SC and 0.6% (1/160) for LM treatment plans. The AD treatment plan was chosen over the SC plan 100% of the time. Examples of SC, AD, and LM treatment plans for a single fraction from 1 patient, along with the associated planning workflow, are shown in Fig. 4.

### Toxicity

Four of 8 patients experienced acute genitourinary (GU) and/or gastrointestinal (GI) toxicity, graded per the National Cancer Institute Common Terminology Criteria for Adverse Events version 5.0 criteria. One patient had both GI (grade 1) and GU (grade 2) toxicity, whereas 3 others had isolated GU toxicity (2 with grade 1 and 1 with grade 2). No grade 3 or 4 toxicities were observed. These adverse effects were transient and resolved with supportive care.

## Discussion

For this study, we presented our initial institutional experience with treating MIBC using CBCT scan guided oART. AD treatment plans were selected for all treatments, encompassing 160 fractions delivered across 8 patients. The AD treatment plan was significantly superior for most target and OAR metrics, except for PTV_high versus the LM treatment plan and rectum_V30 versus the SC treatment plan, where no significant differences were observed. Target and OAR planning objectives were met for 62.3%, 55.4%, and 91.2% of SC, LM, and AD treatment plans, respectively. This suggests that the AD treatment plan approach may increase the therapeutic ratio by covering necessary treatment volumes with the appropriate dose while reducing the dose to OARs. However, clinical trials are needed to correlate these trends with toxicity reductions. DVHs in Fig. 3 illustrate the OAR exposure patterns for the bowel and rectum, with median dose-volumes consistently lower than those in the SC and LM treatment plans. Ethos automatically generates a non-AD treatment scheduled plan for each fraction, which served as an objective benchmark in our analysis. Although a 7 mm PTV margin without adaptation would likely lead to undercoverage, including these plans helped quantify what would have been delivered without real-time adaptation.

Other studies have assessed the feasibility and dosimetric impact of CBCT scan guided oART for MIBC. Åström et al^4^ reported reductions in the median primary PTV volume, bowel bag_V45, and rectum_V50 of 33.0%, 18.8%, and 70.7%, respectively. In their study, over 30% of patients did not receive oART because of workflow limitations and the timing of the COVID-19 pandemic. In contrast, oART was used for every fraction of all patients here. The treating physician/AD therapy physicist selected the oART plan solely, confirming improved dosimetry with oART. In our study, the bladder volume differed from the volume at CT scan simulation by a mean of 60 cc, with most volumes being smaller than those at simulation. The median bowel_V45 was 10 cc for SC treatment plans and 7 cc for AD treatment plans, indicating a 33% reduction with the AD treatment plan. Although the AD and SC treatment plans had similar median rectum means (20 and 21, respectively), nearly 25% of the SC treatment plans had rectal means exceeding the maximum rectal mean in the AD treatment group. Another difference from Åström et al^4^ is our use of simultaneous integrated boost (SIB) for over 50% of patients. All objectives were significantly improved with the AD treatment plans compared with the SC treatment plans, except for rectum_V30. Without SIB, rectal and bowel dosimetry in LM treatment plans would likely be worse, even with the AD treatment plan. These findings support that adaptation generally improves OAR dosimetry, including with SIB.

Standardized PTVs of 1.5 cm are commonly used in bladder-conserving RT to account for bladder size variability.^14^ Pertinent dosimetric endpoints between LM and AD treatment plans have not been directly compared with oART treatment. Although PTV coverage was similar between the 1.5 cm margins in LM treatment plans and 0.7 cm margins in AD treatment plans, the median bowel_V45 for the LM treatment plan was 32 cc. Therefore, nearly 5 times as much bowel received 45 Gy with LM treatment plans compared with AD treatment plans. The median rectum mean for the LM treatment plan was 26 Gy, with nearly 50% of the LM treatment plan cases having rectal means exceeding the maximum mean in AD treatment plans. Although SIB RT improves OAR dosimetry, it remains underutilized in US radiation oncology, making our LM treatment plans representative of current practice.^15^

Patients who received ENI were excluded from our study. Azzarouali et al^5^ used a similar oART CT scan guided SIB approach for MIBC, although all patients in their study received ENI. Fiducial markers guided high-dose volumes in their study, with a median shift of 7.8 mm. Given our 7 mm margin and use of oART with SIB, fiducials may be unnecessary. In our study, PTV D98% exceeded 5225 cGy in 98.1% of fractions in the AD treatment plan arm.

Our clinic’s median AD treatment planning time was 8.9 minutes, and the median setup to treatment completion time was 24.7 minutes. Azzarouali et al^5^ reported a similar total treatment time of 30 minutes. Magnetic resonance (MR) guided oART may improve target visibility with SIB, although it can extend total treatment time by at least 10 minutes.^16^ One MR guided rectal cancer study had treatment times as long as 111 minutes.^17^ In our study, CBCT scan was used for oART, and IMRT was selected over volumetric modulated arc therapy (VMAT) due to faster plan optimization. Although VMAT is used selectively in our clinic when there is a clear clinical advantage, prior studies have shown that Ethos-generated IMRT treatment plans can outperform VMAT treatment plans dosimetrically in both thoracic and pelvic sites.^18^^,^^19^ The median time from CT scan simulation to treatment initiation in our cohort was 26 days, which exceeds the typical 2-week benchmark. We attributed this extended interval to our institution’s initial learning curve associated with implementing oART for MIBC using Ethos.

Verification CBCT scan ensures accuracy in AD treatment plan volume delineation but does not account for intrafraction bladder volume changes. Because the time from verification CBCT scan to treatment delivery increases, bladder volume increases, leading to larger bladder volumes than initially planned. Lalondrelle et al^6^ quantified intrafraction bladder volume increases, observing an average postvoid volume increase of 9 cc over a mean time of 13 minutes, corresponding to 2 to 3 mm of bladder displacement. Similarly, Grønborg et al^20^ reported a median bladder volume increase of 11 cc over 10 minutes during treatment. In our study, we evaluated daily bladder volume differences at CBCT scan compared with simulation, with the 7 mm PTV margin accounting for bladder filling during the AD treatment delivery process.

Despite a small patient cohort and focus on acute toxicity, we observed favorable toxicity profiles with no grade 3 or 4 events and limited grade 2 events, even with concurrent systemic therapy. Historical controls using LM treatment volumes report relatively high levels of acute treatment toxicities. For instance, the BC2001 trial reported ≥ grade 3 toxicity in 36% versus 27.5% of patients in CRT versus RT arms using LM treatment planning.^14^^,^^21^ In contrast, studies have shown a reduction in toxicity when using an AD treatment approach for MIBC. Toxicity data from randomized control trials, RAIDER and HYBRID, demonstrated ≥ grade 3 toxicity in only 3% (non-GU) and 6% of the AD treatment arms, respectively.^22^ Although our study is retrospective, our data support these findings, showing no ≥ grade 3 toxicities, emphasizing that AD treatment planning improves OAR dosimetry and results in limited acute toxicity.

Several strengths of our study should be acknowledged. Patient-specific target volumes and prescriptions were clearly demonstrated, and a robust workflow for oART was implemented for MIBC. All treatments delivered used oART IMRT. A single attending radiation oncologist oversaw AD treatments, directly participating in ∼20% of fractions and reviewing the remainder offline, whereas a consistent team of physicists performed contour edits and plan selection for the remaining fractions—minimizing interphysician variability while circumventing selection bias. Although limited to 8 patients, the analysis includes 160 individual fractions, offering meaningful insight into treatment plan performance.

However, several limitations must also be acknowledged. Single institution design limits replicability and generalizability. Neither fiducials nor MR guidance was used for PTV_ high alignment with SIB RT. Only acute toxicities were reported. Bowel contours > 2 cm superior/inferior to the PTV were inaccurate because of the oART contouring time constraints; only absolute volume metrics were assessed in DVHs.

Future research should involve multiinstitutional collaboration to enhance generalizability and replicability across different settings. Retrospective dosimetric analysis after each fraction with real-time bladder volume monitoring would help account for intrafraction changes, improving dosimetric coverage precision. Further studies incorporating oART and late toxicity follow-up are needed to evaluate sustained treatment effects. Expanding the patient cohort would also increase statistical power, allowing for more detailed analyses and further refinement of oART protocols.

## Conclusion

This study supports that CBCT scan guided oART for MIBC is feasible and can reduce OAR dose exposure while maintaining PTV coverage. Among 8 patients/160 fractions, AD treatment plans achieved superior target coverage and significantly reduced dose to the bowel and rectum compared with SC and LM treatment plans, with reduced PTV margins compared with LM treatment plans. Acute toxicity was minimal and manageable. Using oART for MIBC enhances the therapeutic ratio, ensuring adequate treatment coverage while minimizing OAR doses. Future patient-centered research should investigate the clinical impact of reduced margins of oART in MIBC treatment.
